# Differential susceptibility to colorectal cancer due to naturally occurring gut microbiota

**DOI:** 10.18632/oncotarget.5604

**Published:** 2015-09-10

**Authors:** Aaron C. Ericsson, Sadia Akter, Marina M. Hanson, Susheel B. Busi, Taybor W. Parker, Rebecca J. Schehr, Miriam A. Hankins, Carin E. Ahner, Justin W. Davis, Craig L. Franklin, James M. Amos-Landgraf, Elizabeth C. Bryda

**Affiliations:** ^1^ Rat Resource and Research Center, University of Missouri, Columbia, MO, USA; ^2^ MU Metagenomics Center, University of Missouri, Columbia, MO, USA; ^3^ Department of Veterinary Pathobiology, University of Missouri, Columbia, MO, USA; ^4^ College of Veterinary Medicine, University of Missouri, Columbia, MO, USA; ^5^ MU Informatics Institute, University of Missouri, Columbia, MO, USA; ^6^ Department of Health Management and Informatics, University of Missouri, Columbia, MO, USA

**Keywords:** gut, microbiota, colorectal cancer, Pirc, rat

## Abstract

Recent studies investigating the human microbiome have identified particular bacterial species that correlate with the presence of colorectal cancer. To evaluate the role of qualitatively different but naturally occurring gut microbiota and the relationship with colorectal cancer development, genetically identical embryos from the Polyposis in Rat Colon (Pirc) rat model of colorectal cancer were transferred into recipients of three different genetic backgrounds (F344/NHsd, LEW/SsNHsd, and Crl:SD). Tumor development in the pups was tracked longitudinally via colonoscopy, and end-stage tumor burden was determined. To confirm vertical transmission and identify associations between the gut microbiota and disease phenotype, the fecal microbiota was characterized in recipient dams 24 hours pre-partum, and in Pirc rat offspring prior to and during disease progression. Our data show that the gut microbiota varies between rat strains, with LEW/SsNHsd having a greater relative abundance of the bacteria *Prevotella copri*. The mature gut microbiota of pups resembled the profile of their dams, indicating that the dam is the primary determinant of the developing microbiota. Both male and female F344-Pirc rats harboring the Lewis microbiota had decreased tumor burden relative to genetically identical rats harboring F344 or SD microbiota. Significant negative correlations were detected between tumor burden and the relative abundance of specific taxa from samples taken at weaning and shortly thereafter, prior to observable adenoma development. Notably, this naturally occurring variation in the gut microbiota is associated with a significant difference in severity of colorectal cancer, and the abundance of certain taxa is associated with decreased tumor burden.

## INTRODUCTION

Colorectal cancer (CRC) is a multifactorial disease induced via multiple genetic factors such as the mutations associated with Familial Adenomatous Polyposis (FAP) and Hereditary Non-Polyposis Colorectal Cancer (HNPCC), and a wide range of other factors such as intestinal inflammation, age, diet, alcohol and tobacco consumption, stress, obesity, and activity level, among others. Many of these factors influence the composition of the resident gut microbiota (GM) [1-5], raising the question of whether the GM may serve as a common mediator in pathways through which these factors exert their influence. Moreover, the recently proposed concept of an “etiologic field effect”, referring to an increased risk of polyp formation and subsequent neoplastic transformation in tissue adjacent to resected tumors, may be partially explained by the continued presence of tumorigenic, or absence of protective, microbial factors. Similarly, the relatively strong evidence for heritability of CRC and paucity of loci identified in genome-wide association studies of CRC [6] may be a reflection of the variation in contribution of non-host, i.e., microbial, factors. Thus while familial predisposition to CRC may be a consequence of unidentified modifier loci, it may also be related to characteristics of the GM which are transmitted maternally, and maintained under the influence of genetically determined factors which shape the composition of the GM.

In one of the first studies using culture-independent methods to compare the GM of patients diagnosed with CRC and healthy controls, an association was demonstrated between the incidence of CRC and increased overall microbial diversity [7]. Of note, the authors also found changes in the GM of individuals diagnosed with pre-neoplastic polyps, suggesting that differences or changes in the composition of the GM occur prior to neoplastic transformation. Additionally, the microbiota associated with cancerous tissue exhibits decreased diversity relative to tissue distal to the tumor [8], implying a local effect of the tumor or host response to the tumor capable of modulating the GM. Thus, longitudinal studies incorporating early time points prior to disease development are critical to investigations of the GM in CRC. Considering the extended time course over which CRC develops in humans, such studies necessitate the use of animal models.

Several genetically driven mouse models have been used to demonstrate that the mere presence of GM increases tumor burden [9-12]. Mice carrying mutations in the adenomatous polyposis coli (*APC*) gene (*Apc*^+/min^ mice) serve as a model for both sporadic and familial CRC and studies using dietary modifications suggest that the phenotype of this model is malleable owing to the influence of the GM [13]. For reasons that remain unclear however, mice develop neoplasia almost exclusively in the small intestines. Additionally, studies using *Apc*^+/min^ mice have yielded contradictory data regarding the effect of the GM on tumor burden [11, 14]. The Polyposis in Rat Colon (Pirc) rat model of CRC carries a mutation in the same gene but mirrors the human disease more faithfully with tumor development localized primarily to the colon [15].

Few studies have examined the influence of differences in naturally occurring, complex GM on the initiation and development of CRC. To test the hypothesis that there are naturally occurring compositions of the GM which predispose or protect a host from CRC, the Pirc rat model of CRC was rederived into surrogate dams harboring qualitatively different GM compositions. The resulting genetically identical pups acquired the GM of their respective dams and maintained that GM composition throughout life. A decreased tumor burden was observed in male rats born to Lewis dams, relative to males born to F344 or SD dams, and tumor burden was correlated with the relative abundance of several bacterial taxa present at different levels in the various GM profiles. A similar trend was observed in female rats colonized with the Lewis- and SD-derived GM. These data provide a robust, longitudinal characterization of the gut microbiota during the development of CRC, as well as direct evidence that differences in a naturally occurring GM can significantly affect disease outcomes in genetically susceptible hosts.

## RESULTS

### The gut microbiota of Lewis rats differs from that of F344 and SD rats

**Figure 1:**
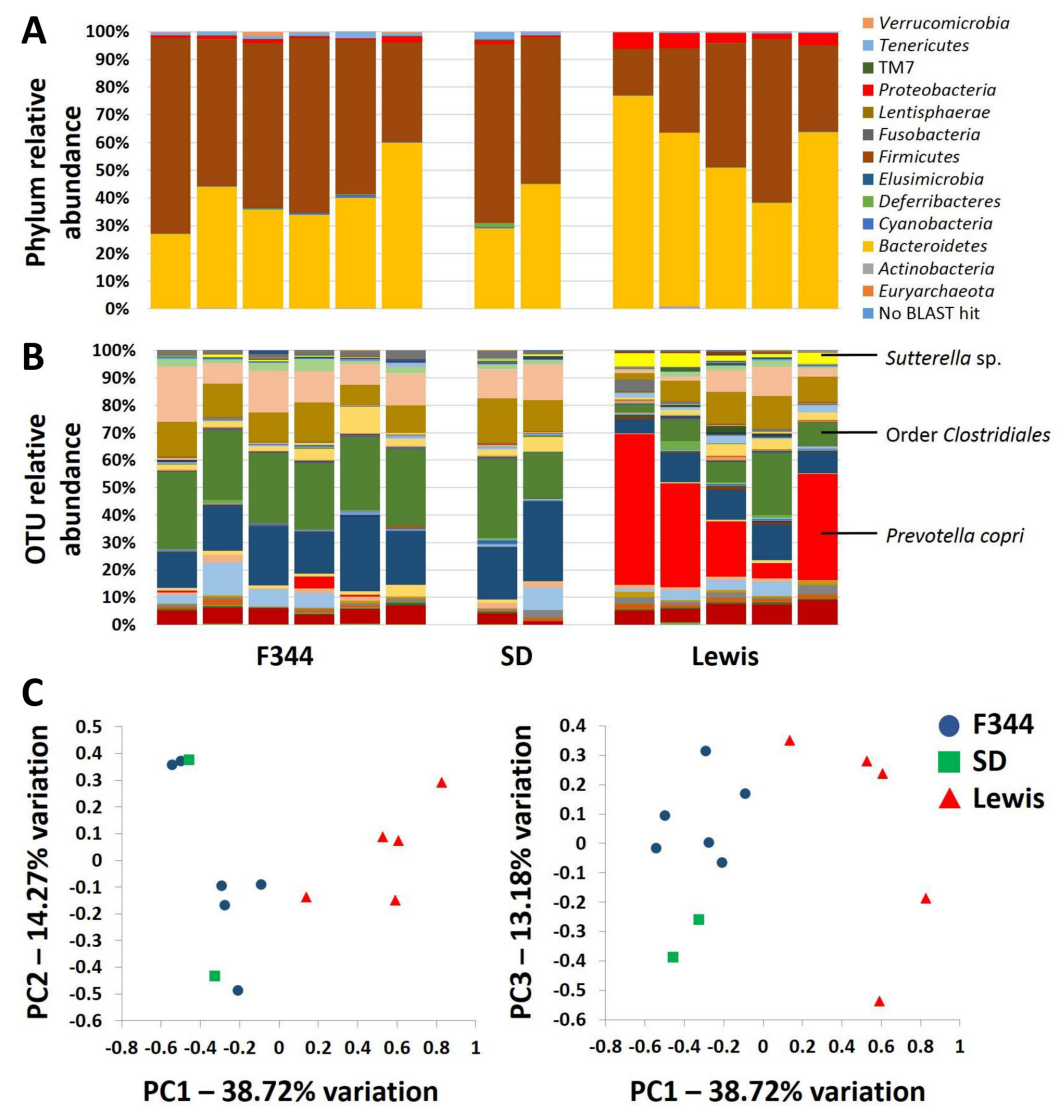
The gut microbiota of Lewis rats differs qualitatively from that of F344 and SD rats Bar charts showing the relative abundance of microbes in the gut microbiota of F344 (*n* = 6), SD (*n* = 2), and Lewis (*n* = 5) surrogate dams 24 hours pre-partum, annotated to the level of phylum (**A**. legend at right) and operational taxonomic unit **B.**; Principal component analysis of the samples depicted in A and B, showing complete separation of Lewis dams from F344 and SD dams along principal component 1 **C**.

To characterize the gut microbiota (GM) present in each genetic background, fecal DNA was extracted, the microbial V4 region was amplified via PCR, and 16S rRNA amplicon sequencing was performed. The resulting microbial profiles differed substantially between Lewis dams and the other genetic backgrounds tested, Fisher (F344) and Sprague-Dawley (SD) rats. Annotation to the level of phylum revealed substantial variability in the relative abundance of the two primary phyla, *Firmicutes* and *Bacteroidetes*, in the GM of Lewis rats, while the GM of F344 and SD rats were similar to each other and showed less variability (Figure 1A). The mean (± st. dev.) of *Firmicutes*:*Bacteroidetes* ratios among Lewis (*n* = 5) dams was 0.73 (± 0.52), as compared to 1.56 (± 0.68) in F344 (*n* = 6) and 1.70 (± 0.74) in SD (*n* = 2) dams (*p* = 0.097, one way ANOVA). There was also a significant difference in the relative abundance of Proteobacterial species between GM profiles (*p* = 0.005, Kruskal-Wallis one way ANOVA on ranks), with Lewis harboring the greatest levels. Pairwise comparisons performed using Dunn's method however failed to detect significant differences. Resolved to the level of operational taxonomic unit (OTU), there was considerable variability among the GM of Lewis dams, while the GM of F344 and SD dams was fairly consistent between rats (Figure 1B). Subjectively, Lewis rats harbored a greater relative abundance of *Prevotella copri* and *Sutterella* sp., and lower relative abundance of microbes in the order *Clostridiales*, as compared to F344 and SD rats. Notably, up to 56.4% of the sequences detected in one Lewis dam were annotated to *P. copri* (mean ± st. dev. 27.93 ± 22.92%). In contrast, *P. copri* constituted between 0.002% and 4.141% (mean ± st. dev. 0.81 ± 1.65%) among the F344 and SD dams. Principal component analysis (PCA) showed clustering of the F344 and SD samples distinct from that of the Lewis fecal samples (Figure 1C). Notably, Lewis and F344 surrogate dams were purchased from Harlan Laboratories while SD dams were purchased from Charles River, suggesting that the GM composition is not a function of the vendor from which the rats were obtained.

### Rederivation of F344-Pirc rats via embryo transfer in F344, SD, and Lewis surrogate dams

In order to generate isogenic mutant rats harboring different GM, F344-Pirc embryos generated from mating a wild-type F344/NTac female to a heterozygous (F344/NTac-*Apc*^+/am1137^) male (Supplementary Figure S1A) were transferred into F344, SD, or Lewis surrogate dams. Weanling pups were separated by sex and genotyped to identify F344-Pirc males; we opted to focus on males as, historically, they develop greater tumor burdens than females [16]. Following five rounds of embryo transfer (ET) into surrogate dams of each genetic background, eight heterozygous male F344-Pirc pups were born to both F344 and Lewis dams, and five heterozygous male F344-Pirc pups were born to SD dams (Supplementary Table 1). After multiple additional rounds of ET into SD dams resulting in no male pups carrying the desired genotype, efforts were discontinued, resulting in final sample sizes of eight male F344-Pirc rats colonized with GM_F344_, eight with GM_Lewis_, and five with GM_SD_. Additionally, seven and four heterozygous female rats harboring GM_Lewis_ and GM_SD_, respectively, were generated. Supplementary figure S1B shows the time points for sample collection from the male rats; only endpoint analyses were performed on female rats.

### Rederived pups acquire the gut microbiota of their surrogate dam

**Figure 2:**
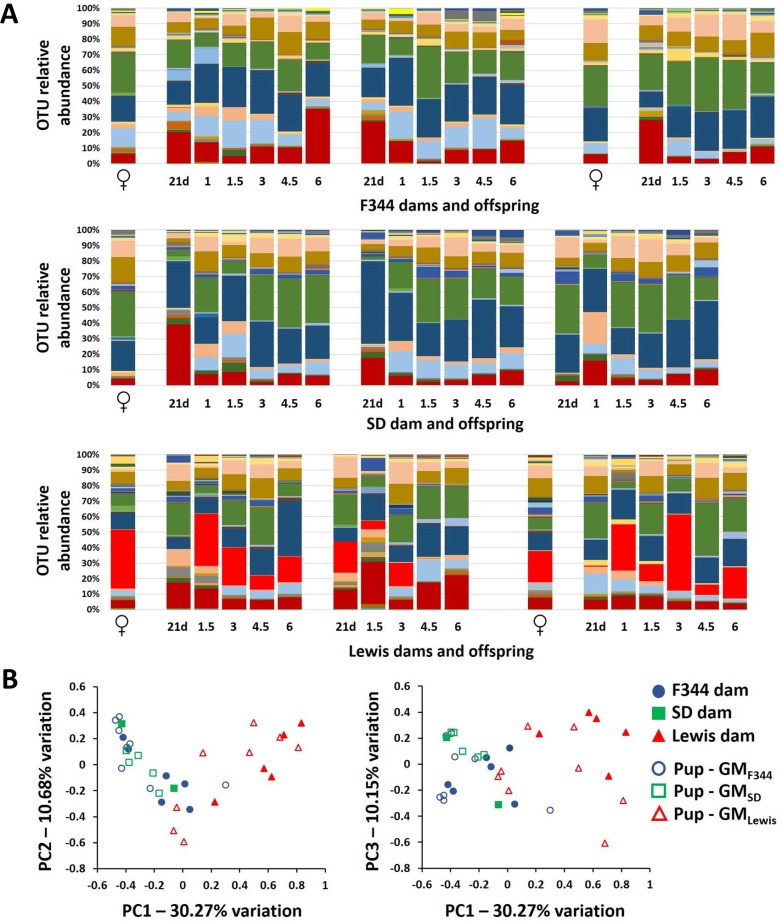
Rederived pups acquire the gut microbiota of their surrogate dam Bar charts showing the OTU relative abundance of representative F344, SD, and Lewis dams and three F344-Pirc pups born to those dams at weaning (21 days), 1 month (1m), 1.5m, 3m, 4.5m, and 6m of age **A.** Principal component analysis (PCA) of all surrogate dams (*n* = 13) and all male F344-Pirc pups born to those dams (*n* = 21) at 1.5 months of age (**B.** legend at right).

Like most mammals, rat pups are seeded with the GM of their birth dam. However, host genotype is also a strong determinant of the composition of the GM. To determine whether rederived F344-Pirc rats developed and maintained a GM consistent with the dam that delivered them, feces were collected and analyzed at multiple time points. The GM of the rederived rats showed considerable temporal variability but an overall similarity to that of the dam that delivered and nursed them (Figure 2A). Specifically, *P. copri* was detected in pups born to Lewis dams at levels similar to that found in their surrogate dams and at much lower levels in pups born to F344 and SD surrogate dams. Accordingly, the GM of F344-Pirc rats at 1.5 months of age clustered with samples from their surrogate dam on day 20 of gestation, providing evidence that the F344-Pirc rats were colonized with the GM of their dam (Figure 2B). Hierarchical clustering analysis of the GM of male pups at all time points demonstrated a similar separation between the three GM profiles (Supplementary Figure S2), confirming that the offspring harbored distinct GM communities, acquired from the surrogate dams.

### Male F344-Pirc rats with GM_Lewis_ develop less tumor burden than rats with GM_F344_ or GM_SD_

**Figure 3:**
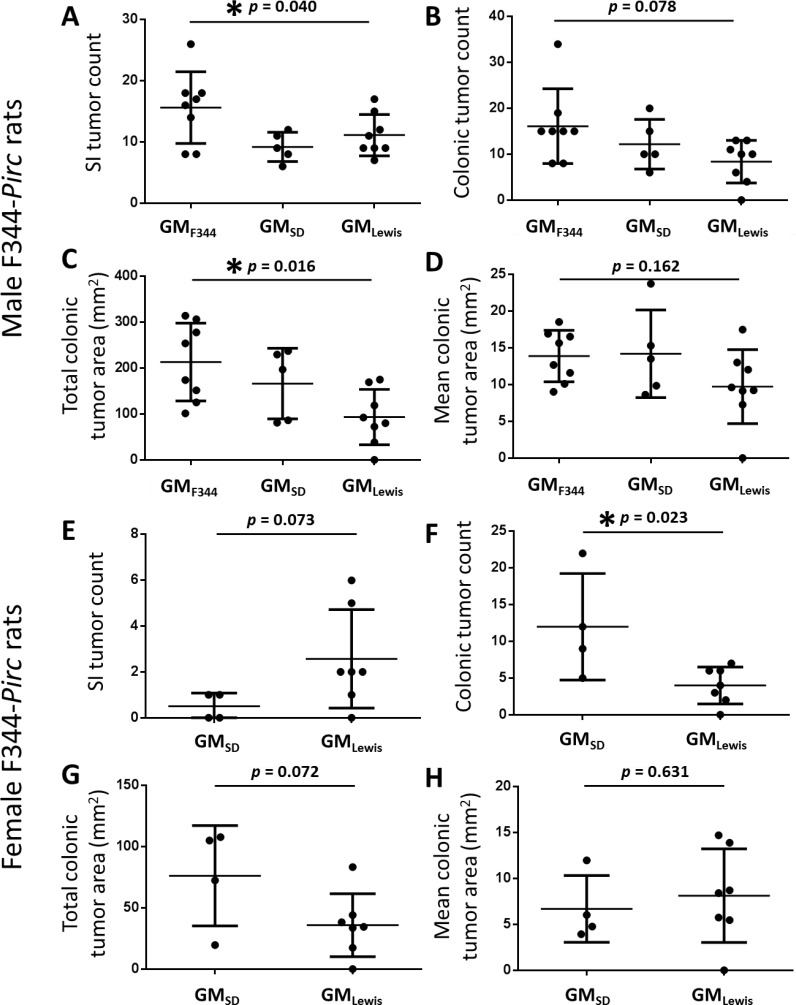
Rats colonized with GM_Lewis_ develop less severe disease than rats harboring GM_SD_ or GM_F344_ Dot plots demonstrate the mean (± SD) small intestinal (SI) tumor count (**A.**, **E.**), colonic tumor count (**B.**, **F.**), total colonic area affected by tumors (**C.**, **G.**), and mean colonic area of individual tumors (**D.**, **H.**) in male (A-D) and female (E-H) F344-Pirc rats colonized with the GM acquired from their cognate surrogate dam. P values denote results of one way ANOVA (males) or t-test (females) and asterisks indicate *p* ≤ 0.05.

To determine endpoint tumor burden, rats were euthanized at 6 months of age and tumors were counted throughout the entire small and large intestines under a dissecting microscope; colonic tumor area was calculated using digital images. In general, a greater tumor burden was detected in both the small and large intestines of male rats colonized with the GM_F344_, relative to male rats colonized with the other GM profiles. Tumor counts in the small intestines ranged from 8 to 26 (mean 15.6), from 6 to 12 (mean 9.2), and from 7 to 17 (mean 11.1) in rats harboring GM_F344_, GM_SD_, and GM_Lewis_, respectively (Figure 3A). One-way ANOVA detected a significant difference between all groups (*p* = 0.040) although pairwise comparisons using the Holm-Sidak method failed to achieve significance (GM_F344_ vs. GM_SD_, *p* = 0.056). Similarly, colonic tumor counts suggested a detrimental effect of the GM_F344_ with 8 to 34 (mean 16.1), 6 to 20 (mean 12.2), and 0 to 13 (mean 8.4) tumors being detected in the colon of rats colonized with GM_F344_, GM_SD_, and GM_Lewis_, respectively (Figure 3B). Testing via ANOVA detected no significant difference in the number of colonic (or total) tumor counts (*p* = 0.078 and 0.064, respectively) although there was a clear trend toward a protective effect of GM_Lewis_. When rats harboring GM_SD_ were removed from the analysis, t-test detected a significant difference between rats with GM_F344_ and GM_Lewis_ in both colonic and total tumor count (*p* = 0.021 and 0.043, respectively). Of note, historical tumor counts in non-rederived Pirc rats on the F344/NTac genetic background most closely resemble that seen in Pirc rats rederived in either F344/NHsd or SD surrogate dams, suggesting a protective effect of the GM_Lewis_, as opposed to a detrimental effect of the other GM compositions.

Total colonic area affected by tumor varied from 0 mm^2^, in a rat colonized with GM_Lewis_ in which no colonic tumors were detected, to 314 mm^2^ in a rat colonized with GM_F344_. The male rat harboring GM_Lewis_ in which no tumors were detected was of particular interest as it represented the first F344-Pirc rat in a historical dataset of over 200 male F344 rats to develop no colonic tumors. Accordingly, genomic DNA was extracted and genotyped a second time confirming that it was heterozygous for the Pirc mutation. Despite the modest sample size, statistical testing detected a difference in the total colonic tumor area between groups (*p* = 0.016) with rats harboring GM_F344_ developing more wide-spread disease than those harboring GM_Lewis_; rats colonized with GM_SD_ demonstrated an intermediate phenotype (Figure 3C). There was no difference in the mean colonic tumor area, i.e., the total tumor area divided by the number of colonic tumors, between groups although there was again a trend for decreased tumor growth in rats colonized with GM_Lewis_ (Figure 3D; *p* = 0.162).

### Female F344-Pirc rats colonized with GM_Lewis_ are similarly protected

During rederivation of the F344-Pirc rats, heterozygous female pups colonized with GM_SD_ and GM_Lewis_ were also generated; no heterozygous female pups were born to F344 dams. As in the rederived male pups, female pups harbored GM profiles very similar to that of their surrogate dam, and variation between individuals was dominated by differential abundance of *P. copri* and unresolved microbes in the family *Prevotellaceae*. Tumor counts and measurements at 6 months of age revealed a similar protective effect of the GM_Lewis_ with female F344-Pirc pups born to Lewis dams demonstrating a trend for fewer tumors overall and significantly fewer colonic tumors than pups born to SD dams (t-test; *p* = 0.023)(Figure 3E, 3F). As in the rederived male rats, one female F344-Pirc rat colonized with GM_Lewis_ failed to develop colonic tumors. Differences in total tumor count (t-test; *p* = 0.119) and colonic area occupied by tumor (t-test; *p* = 0.072) showed similar trends (Figure 3E, 3G). There was no difference between GM profiles in the mean colonic tumor area in females (Figure 3H).

### Variation between the Lewis and F344/SD gut microbiota is driven by family *Prevotellaceae*

**Figure 4:**
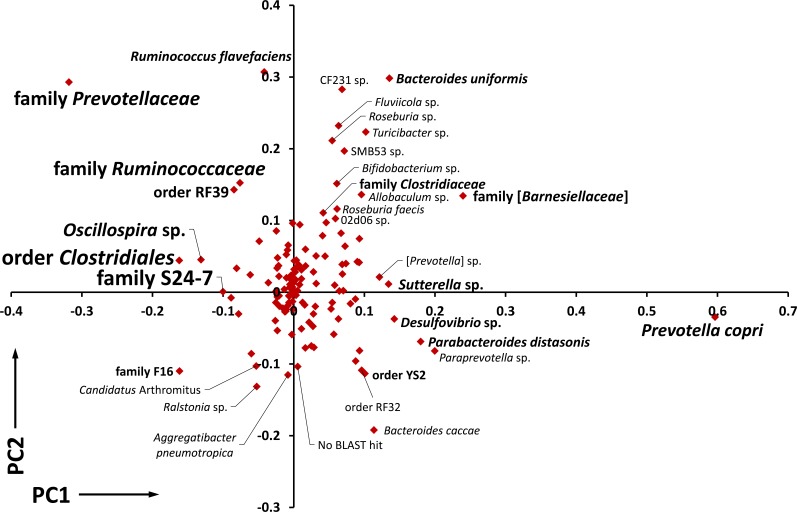
Contribution of individual OTUs to the variation between GM profiles Loadings plot of the PCA data depicted in Figure 2, showing the relative contribution of all operational taxonomic units (OTU) to principal component 1 (PC1) and PC2, OTUs located greater than 0.1 eigenvalue from the origin are labeled with font size corresponding to overall abundance in the dataset.

To determine which taxa were making the greatest contribution to the variability between the three GM profiles, a loadings plot was generated from the same data matrix used to construct the PCA shown in Figure 2. Loadings plots are constructed from column vectors in the data matrix, rather than row vectors, as a means to interpret the relationships seen in the latter. Based on the clear partitioning of the Lewis-derived GM (GM_Lewis_) from GM_F344_ and GM_SD_ along principal component 1 (PC1), the microbes contributing to that separation were of greatest interest. Clearly, *P. copri* and other unresolved members of the family *Prevotellaceae* contributed the greatest variability to the data set (Figure 4). Loadings plots constructed of samples collected at other time points, or using a combination of all time points, showed similar plots with *P. copri* and *Prevotellaceae* lying at opposite ends of PC1.

To determine if the differences between GM_Lewis_, GM_F344_, and GM_SD_ are temporally stable, and to characterize the progression of the GM prior to and during the development of CRC, microbial profiles at each time point were tested for differences in α-diversity, as well as the relative abundance of specific bacterial families associated with an altered risk of CRC. Regarding α-diversity as determined via the Chao1 and Shannon diversity indices (Supplementary Figure S3), while all groups showed a similar trend over time, no significant differences were detected between the three GM profiles at any time point.

**Figure 5:**
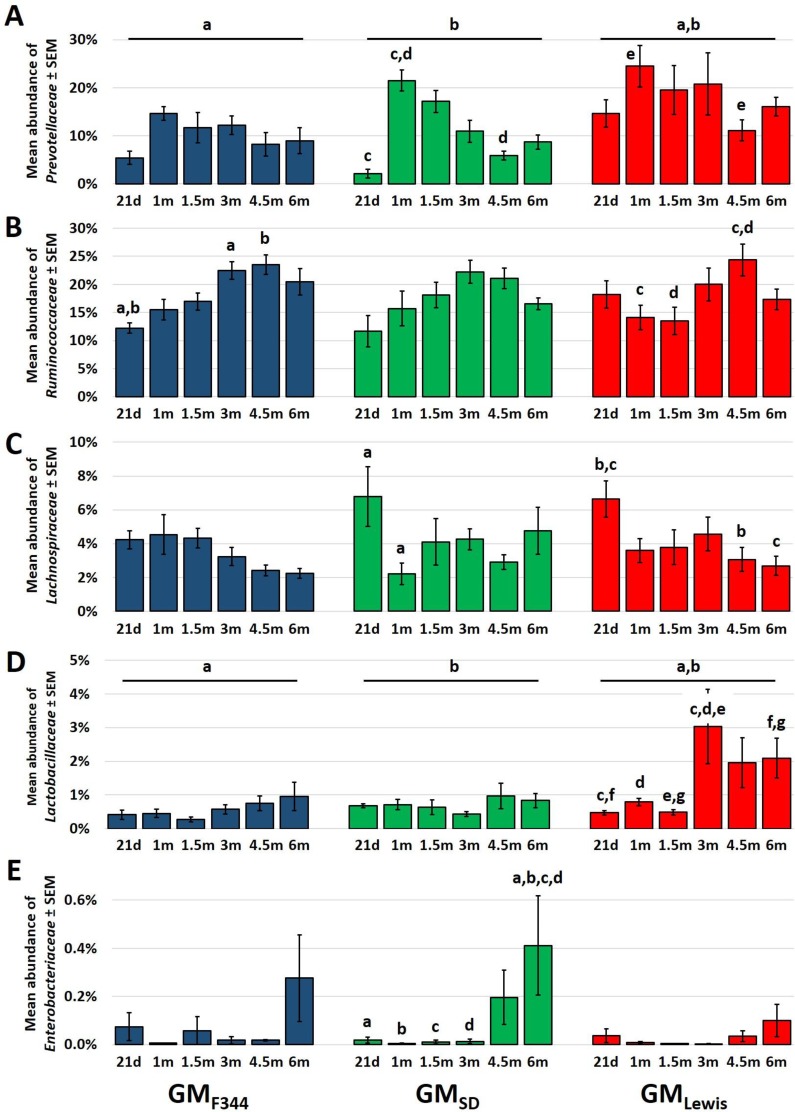
The Lewis GM undergoes a progression distinct from that of F344 or SD Bar charts demonstrating the progression in terms of relative abundance (mean ± SEM) of *Prevotellaceae* **A.**, *Ruminococcaceae* **B.**, *Lachnospiraceae* **C.**, *Lactobacillaceae* **D.**, and *Enterobacteriaceae* **E.** at 21 days (21d: weaning), 1 month (1m), 1.5m, 3m, 4.5m, and 6m of age in isogenic male F344-Pirc rats colonized with GM_F344_ (*n* = 8), GM_SD_ (*n* = 5), and GM_Lewis_ (*n* = 7 or 8). Like letters indicate significant (*p* ≤ 0.05) differences; 2-way ANOVA with post hoc pairwise comparisons via the Holm-Sidak method. There was a significant main effect of time on the relative abundance of all taxa analyzed; only significant differences within GM are denoted for the sake of clarity. No significant interactions were detected between GM and age for any taxon analyzed.

Five bacterial families were targeted for testing based on putative effects on relative risk of CRC. Specifically, *in vivo* and *in vitro* data suggest beneficial effects of microbes in the families *Lachnospiraceae*, *Ruminococcaceae*, *Prevotellaceae*, and *Lactobacillaceae* [17], while families such as *Enterobacteriaceae* have been associated with an increased risk of CRC [18, 19]. The mean relative abundance of each family was determined at weaning (21d; 21 days of age), one month (1m), 1.5m, 3m, 4.5m, and 6m of age and two-way ANOVA was performed with GM (i.e., F344, SD, or Lewis) and age as independent variables. Data were first analyzed to identify interactions (i.e., a differential effect of one variable dependent on the level of the other variable) and no significant interactions were detected between GM and age in the abundance of any of the five families tested. However, a significant effect of age on the abundance of all families was detected (*p* < 0.001 for *Prevotellaceae*, *Lachnospiraceae*, *Ruminococcaceae*, and *Enterobacteriaceae*; *p* = 0.018 for *Lactobacillaceae*), with certain taxa assuming relatively consistent progressions from weaning to 6m (Figure 5). For example, the proportion of *Ruminococcaceae* gradually increased with age in all three GM communities and then decreased, albeit not significantly, between 4.5m and 6m (Figure 5B). Similarly, the relative abundance of *Enterobacteriaceae* was relatively stable in all three GM until later in the disease course but then increased in all three GM to varying degrees (Figure 5E). Significant main effects of GM were detected in the relative abundance of *Prevotellaceae* and *Lactobacillaceae*, with GM_Lewis_ containing greater proportions of both families relative to the other GM profiles (Figure 5A and 5D). Of note, there was an apparent difference between GM_Lewis_ and the other GM profiles in the trajectory of *Lactobacillaceae* and *Enterobacteriaceae* at the later time points. Specifically, F344-Pirc rats harboring GM_Lewis_ experienced a significant, sustained increase in the relative abundance of *Lactobacillaceae* at 3m and only a slight increase in the amount of *Enterobacteriaceae* detected, whereas rats colonized with GM_F344_ and GM_SD_ demonstrated a reciprocal shift, i.e., an increase in *Enterobacteriaceae* (significant in GM_SD_ at 6m with a similar trend in GM_F344_) with no concomitant increase in *Lactobacillaceae* (Figure 5D and 5E). These data demonstrate that the GM_Lewis_ underwent a temporal progression distinct from that of GM_F344_ and GM_SD_ favoring putatively beneficial microbes.

It is also of interest what influence the Pirc genotype *per se* may have on the GM over time, relative to wild-type littermates. To evaluate any such potential genotype-associated effects on the GM, fecal samples from non-rederived male and female F344-Pirc littermates of each genotype were collected one week after weaning (at 1 month of age) and again at 4 months of age, and characterized as above. Surprisingly, there was greater variability detected between samples collected at 1 and 4 months of age than between wild-type and heterozygous littermates, in both male (Supplementary Figure S4) and female (Supplementary Figure S5) rats, suggesting that the naturally occurring changes in the composition of the GM over time outweigh any genotype-dependent changes. Thus, while genotype and the composition of the GM both appear to influence susceptibility to CRC, no indirect genotype-dependent influence on the GM was detected.

### Associations between specific taxa and tumor burden

To elucidate any potential relationships between microbial taxa and the initiation or progression of tumor development, testing for correlations was performed. Tumor data (mean and total tumor areas, colonic and total tumor counts) were compared to the normalized relative abundance of all consistently detected OTUs. As any detected associations between tumor burden and the relative abundance of OTUs at later time points (4.5m and 6m) could reflect tumor-mediated effects, correlative statistical analysis focused on the early time points up to and including 3m, to identify taxa an increased abundance of which would correlate with an increased or decreased tumor burden at necropsy.

**Figure 6:**
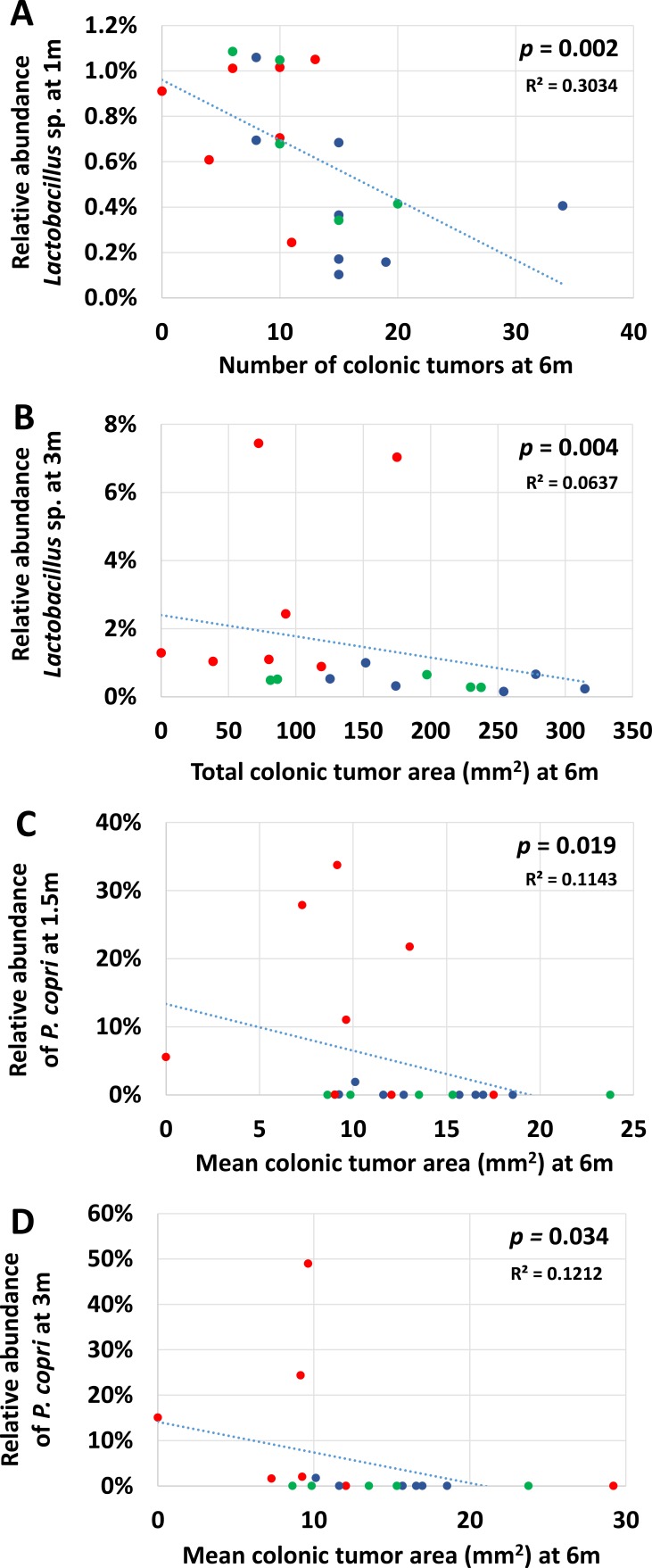
Correlations between relative abundance of select OTUs early in life and terminal tumor burden Scatter plots showing significant negative correlations detected between the relative abundance of *Lactobacillus* sp. at 1m **A.** or 3m **B.** of age and total number of colonic tumors **A.** or total colonic area affected by tumors **B.** at necropsy, or the relative abundance of *P. copri* at 1.5 **C.** or 3m **D.** of age and mean area occupied by individual colonic tumors at necropsy. P-values determined via Spearman rank order correlation.

Perhaps reflecting the combined influence of microbial consortia in any increased or decreased risk of CRC, there were no individual OTUs which consistently correlated with any tumor read-out at all early time points. There were however a small number of OTUs that correlated with tumor count or tumor area at one or more time points, and for whom a physiological role in disease protection could be inferred. Notably, relative abundance of the genus *Lactobacillus* at 1m and 3m correlated negatively with total and colonic tumor counts (*p* = 0.003 and 0.002 respectively at 1m, 0.046 and 0.020 at 3m) (Figure 6A). There was also a significant negative correlation with total colonic tumor area at 3m (Figure 6B, *p* = 0.004) and a similar trend at 1m (*p* = 0.070).

Significant correlations were also detected between the relative abundance of *P. copri* and mean colonic tumor area at 1.5m and 3m (Figure 6C and 6D; *p* = 0.019 and 0.039, respectively), with a similar, albeit insignificant (*p* = 0.084), trend at 1m. There were also trends toward a protective effect of *P. copri* at 1m and 3m with regard to total colonic tumor area (*p* = 0.097 and 0.091, respectively). These findings may reflect the overall disparity in relative abundance of *P. copri* between GM profiles as the correlations failed to achieve significance when data were stratified with GM.

Lastly, the relative abundance at weaning of the microbe *Akkermansia muciniphila*, was found to positively correlate with multiple tumor measures including total colonic tumor area and mean colonic tumor area (*p* = 0.006 and 0.039, respectively). When analysis was stratified within GM_F344_ or GM_Lewis_ however, the association with total colonic tumor area persisted in rats harboring GM_F344_, but not those with GM_Lewis_. Conversely, the association with mean colonic tumor area persisted in rats with GM_Lewis_, but not those with GM_F344_.

There were a handful of other microbes which were detected at very low relative abundance and for whom significant correlations with tumor burden were detected, including *Elusimicrobium* sp., *Coprococcus eutactus*, and *Paraprevotella* sp. That said, these microbes were each detected primarily, or exclusively, in only one of the GM profiles, and no correlations persisted when tested within GM. Overall, the dearth of significant correlations identified between individual OTUs and differential disease severity implies that no single bacterial taxon can explain the observed differences. More likely, any differential disease susceptibility conferred by the host GM is due to multifactorial differences in the composition of the GM or functional differences in the bacteria present.

## DISCUSSION

Here we show that isogenic individuals residing in controlled environments but harboring distinct GM profiles possess differential susceptibility to CRC. Specifically, we prospectively demonstrate a significant impact in CRC susceptibility due to naturally occurring GM present from birth, and identify correlations between disease severity and characteristics of the GM prior to the development of grossly visible lesions. Prior studies have demonstrated that germ-free (GF) mice or rats are partially protected from CRC, suggesting that the mere presence of commensal microbes contributes to disease development or progression [9, 10, 12, 20]. Similarly, the role of specific microbes, including *Escherichia coli*, *Enterococcus faecium*, *Bifidobacterium* spp., *Lactobacillus acidophilus*, *Clostridium* spp., and others, has been investigated using mono-associated mice and rats, with varying results [18, 21-26]. Such studies provide compelling evidence that the composition of the GM is capable of influencing the disease phenotype. That said, studies of GF and mono-associated animals do not take into account the cross-feeding and competition that occurs in complex microbial syntrophies, and few studies have examined differences in disease susceptibility between subjects harboring distinct, naturally acquired GM profiles. Using transfer of genetically identical embryos into surrogate dams harboring distinct microbial communities, we were able to generate isogenic rats that were carried to term, delivered, and colonized by the respective maternal GM in the normal, physiological fashion. It is worth noting that the colony serving as the basis for these studies was previously rederived (> 3 years ago) using embryo recipients of the same genetic background as the donors, i.e. F344/NTac. The model phenotype was monitored closely following rederivation and no differences were detected in tumor multiplicity or size, relative to historical data prior to rederivation.

In humans, there is considerable variability in the microbes found to positively correlate with CRC in affected individuals when compared to healthy controls [7, 27-30], reflecting the influence of genetic and environmental factors. However, certain characteristic changes have been found repeatedly in fecal samples collected from patients diagnosed with colorectal cancer suggesting a mechanistic link between bacteria and cancer presence, and potentially recurrence risk. These include increased relative abundance of microbes in the families *Fusobacteriaceae* [8, 31-33] and *Bacteroidaceae* [27, 28, 33], and decreased relative abundance of butyrate-producing microbes from the families *Ruminococcaceae* and *Lachnospiraceae* such as *Faecalibacterium* sp., *Roseburia* sp., *Blautia* sp., *Eubacterium* sp., and others [8, 27, 30, 33-35]. One possible mechanism by which butyrate confers protection against CRC is its potent inhibition of histone deacetylase activity [36, 37]. In the current data set, there was a trend toward greater relative abundance of the family *Ruminococcaceae* in the GM_Lewis_ when compared to GM_F344_ and GM_SD_ at weaning, although this did not achieve statistical significance. In contrast, there was a consistent difference between GM_Lewis_ and the other GM in the abundance of the family *Prevotellaceae*, particularly the microbe *P. copri*. Notably, Weir et al. reported a 40-fold greater abundance of *P. copri* in the stool of healthy controls relative to samples from 10 patients recently diagnosed with CRC [30]. Lending further support for a beneficial role of *P. copri* in intestinal health, Kang et al. reported a decreased relative abundance of several unresolved *Prevotella* spp. closely related to *P. copri* in the GM of autistic children experiencing adverse gastrointestinal symptoms [38].

The associations seen in the present study between the relative abundance of *P. copri* and mean colonic tumor area and number suggest a suppressive effect on tumor growth. Since its first isolation from human stool, surveys of the human gut microbiome have revealed that *Prevotella* spp., and *P. copri* in particular, are highly prevalent in the GM of healthy individuals and may in fact represent signature taxa in its composition [39]. *Prevotella* spp. in general possess fermentative enzymatic activity against a broad range of polysaccharides and are thus thought to be capable of generating metabolic products such as the short chain fatty acids (SCFA) propionate and butyrate from a wide range of sources. Like butyrate, propionate is a histone deactylase inhibitor capable of repressing inflammatory signals and cell proliferation [36, 37], suggesting a direct mechanistic link with tumor progression. A role for SCFA production in disease resistance to CRC is further supported by the location of the effect (i.e., the colon), as butyrate and propionate exert many of their benefits via colonocyte uptake. It remains to be seen if supplementation of *Prevotella* spp. at later time points can help prevent initiation, progression, or recurrence of disease.

While less likely, it is also possible that the GM_Lewis_ lacks some protumorigenic taxon or metabolic capacity present in the other two GM communities. Multiple groups have demonstrated the mutagenic and tumor-promoting effects of human intestinal bacteria, often via conversion of dietary components to substances capable of generating DNA adducts [40, 41]. Considering however the presence of two animals in the current study that failed to develop colonic tumors, in the context of the authors' large historical dataset of rats (of multiple genetic backgrounds) harboring the Pirc mutation, it is unlikely that the F344-Pirc rats colonized with GM_Lewis_ are uniquely lacking specific taxa responsible for driving carcinogenesis in all other rats studied heretofore.

Regarding the paucity of significant correlations between OTUs at early stages of disease development and eventual disease burden, there are multiple possible explanations. First, the present study was modestly powered owing to the labor and high costs associated with both the rederivation procedures and sequencing. In the absence of those constraints, it is likely that more or stronger associations would be found. Second, the analysis of the GM did not take into account microbial function; similarities in the 16S rRNA profile do not necessarily mean similar metabolic function of the GM. Work from the lab of Turnbaugh demonstrated that similar bacterial populations can have dramatically different metabolic capacities [42]. Lastly, it must be considered that the influence of commensal bacteria on health and disease risk is often a function of the population as a whole, rather than individual species. The “one bug” mentality that has historically been used in the search for pathogens, e.g., Koch's postulates, is insufficient for studies of commensal populations. Future studies will employ whole-genome shotgun sequencing, metatranscriptomic, or metabolomic approaches to interrogate those differences.

Despite the modest sample size used in the current study, significant differences in total colonic tumor area and colonic tumor count were detected in isogenic male and female F344-Pirc rats, respectively, harboring distinct GM profiles. These findings suggest that there may be subtle differences in the GM of otherwise healthy individuals that predispose them to CRC. From a purely practical standpoint, these data also highlight the variable introduced by rederivation of genetically manipulated animal models of disease. It is only a minority of the most commonly used animal models for which live breeding colonies are maintained. The majority of knock-out and transgenic rats and mice are maintained as cryopreserved germplasm and resuscitated through assisted reproduction techniques such as ET into a surrogate dam. In the interest of reproducibility, researchers would be well-advised to collect and store fecal (or other) samples from strains being cryopreserved. Should a change in phenotype be detected upon rederivation, those archived samples can be compared to those of the resuscitated progeny or, alternatively, used to restore the GM to its original composition. Rederivation-associated alterations in the GM may have a profound impact on experimental reproducibility between institutions, especially those requiring rederivation into SPF facilities.

The implications of this study and others highlight the future challenges in identifying host genetic variation responsible for susceptibility to CRC. McKnite and colleagues have shown that host genetics influence the GM in mice, however our data support the hypothesis that the GM can be stably altered in genetically identical animals [43]. This reveals a complex confounding factor for traditional human genome wide association studies wherein GM data are not available. Genetic studies of heritability of CRC have found strong interactions between the host genome and the environment, but only a small fraction can be explained by known factors (e.g., smoking) [44]. The GM may reveal missing heritability and, at least, will provide a measurable variable when examining genome and environmental interactions. Because of the dynamic nature of the GM, there may be an important period in the development of CRC where exposure to certain bacteria is significant. The use of longitudinal collection in this study suggests that early exposure (weaning to 3 months in rats) has a strong influence on later life disease state regardless of GM composition at the time of frank disease.

Collectively, these data provide proof-of-principle that naturally occurring, pathogen free, complex gut microbiota may affect the relative risk of the development of colorectal cancer. The future challenge is to manipulate the existing GM during various life stages to determine the long-term impact on CRC development and growth. With current advancements in anaerobic culturing systems, selective targeted lysis, and longitudinal assessment of colonization, individual species could be manipulated in complex systems leading to truly targeted probiotic preventative treatments of early precancerous adenomata.

## MATERIALS AND METHODS

### Rats

For embryo transfer (ET) recipients, seven to eight week old female Crl:SD (Charles River Laboratories, Wilmington, MA), F344/NHsd, and LEW/SsNHsd (Harlan Laboratories, Indianapolis, IN) rats were purchased and allowed to acclimate for one week prior to use. Sexually mature male F344-*Apc*^+/am1137^ (F344-Pirc) rats [15], bred on site, were mated to eight week old female F344/NTac rats (Taconic, Hudson, NY) and used as embryo donors. Vasectomized, seven to eight week old Crl:SD male rats (Charles River Laboratories) were co-housed with recipient females to induce pseudopregnancy. Rederived male and female F344-Pirc pups were pair-housed by sex, according to the genetic background of their surrogate dam. All rats were housed in microisolator cages on ventilated racks (Thoren, Hazleton, PA) on a 14:10 light:dark cycle, and fed 5058 (irradiated) breeder chow (LabDiet, St. Louis, MO) with *ad libitum* access to acidified autoclaved water. All procedures were performed according to the guidelines set forth by the Guide for the Use and Care of Laboratory Animals, the Public Health Service Policy on Humane Care and Use of Laboratory Animals, and the Guidelines for the Welfare of Animals in Experimental Neoplasia, and were approved by the University of Missouri Institutional Animal Care and Use Committee.

### Embryo collection and transfer

Estrus synchronization for embryo collection from donors or transfer into pseudopregnant recipients was achieved via intraperitoneal (IP) injection of 40 μg luteinizing hormone-releasing hormone (LHRH) (Sigma, St. Louis, MO) on day 0, at 2.5 hours after the beginning of the light cycle. On day 1, donors received IP injection of 20 IU of PG600 (Valley Vet, Marysville, KS) in 0.2 mL Dulbecco's phosphate-buffered saline (DPBS) with no calcium or magnesium (Life Technologies), again at 2.5 hours post-light induction, to induce superovulation. At 5 hours post-light induction of day 3, donors received IP injection of 40 IU human chorionic gonadotropin in 0.2 mL DPBS. On day 4, embryo donors were mated to intact males; embryo recipients were mated with a sterile, vasectomized male. F344-Pirc embryo donors were euthanized four days post-mating and morulae were collected aseptically. Briefly, the peritoneal cavity was opened and the reproductive tract visualized. Oviducts were excised and placed in a 50 μL drop of pre-warmed type IV-S hyaluronidase (Sigma) reconstituted at 1 mg/mL in HEPES media (Sigma) supplemented with 4 mg/mL bovine serum albumin (Sigma) for five to ten minutes. Clutches of oocytes were released from oviducts with gentle manipulation under a dissecting microscope, and collected with a sterile glass hand-pipette.

Synchronized recipient females (F344/NHsd, LEW/SsNHsd, or Crl:SD) were inspected for copulatory plugs and plug-positive rats were used for ET. Briefly, rats were anesthetized via IP injection of ketamine/xylazine at 1 mg and 5.5 mg per 100 g body weight respectively, and placed in sternal recumbency. A dorsal midline incision was made and oviducts were located by dissecting through retroperitoneal muscle. Approximately 6 to 8 zygotes in 3 to 5 μL media were injected directly into the infundibulum on each side using a glass hand-pipette. Skin incisions were closed with sterile surgical staples and rats received subcutaneous injection of 2 mg/mL flunixin (banamine) prior to recovery on a warming pad.

### Genotyping

Approximately 1 mm portions of tail-tip were excised from pre-weanling rats using sterile technique. DNA was extracted using DNeasy Tissue and Blood kits (Qiagen, Valencia, CA), per manufacturer's instructions. High resolution melt (HRM) analysis was used to genotype the Pirc rats. PCR was performed in a reaction volume of 20 μL containing 0.1 μM of each primer (5′-TGTCAGGAAGACGACTATGAAGA-3′ and 5′-CAGAATAACGTTCACTGTAGTTGG-3′), 10 ng/μL genomic DNA and 1× HRM Supermix (BioRad, Hercules, CA). Reactions and analysis were performed with a BioRad CFX96 Real-Time PCR Detection system with the following cycling conditions: 95°C, 2 min; 40 cycles of 95°C, 10 sec; 60°C, 30 sec; 72°C, 30 sec; 95°C, 30 sec; 60°C, 1 min, followed by melt curve analysis from 65°C to 95°C in increments of 0.2°C for 10 sec. Product analysis was performed using BioRad Precision Melt software to detect the A to T transversion in the *Pirc* allele.

### Fecal sample collection

Fecal samples were collected from surrogate dams at 24 hours pre-partum, and from rederived rats at weaning, 1.5 months (1.5m), 3m, 4.5m, and 6m of age. Rats were placed in an empty autoclaved cage and allowed to defecate normally. One fecal pellet per rat per time point was collected aseptically and placed in a 2 mL round-bottom tube containing 800 μL of lysis buffer [45] and a 0.5 cm diameter stainless steel bead (Penn Ball and Sprocket, NJ).

### Fecal DNA extraction

Fecal DNA was extracted and quantified as previously described [45].

### 16S library preparation and sequencing

Bacterial 16S rRNA amplification and sequencing were performed as previously described [45], at the University of Missouri DNA Core facility (Columbia, MO).

### Informatics analysis

Assembly, filtering, binning, and annotation of DNA sequences were performed at the MU Informatics Research Core Facility (Columbia, MO) as previously described [45]. Principal component analyses and loading plots were generated using a non-linear iterative partial least square (NIPALS) algorithm, implemented in a Macro-enabled Excel worksheet kindly provided by Hiroshi Tsugawa of the Riken Institute (Wako, Japan).

### Colonoscopy

Colonoscopies were performed as previously described [46], at 3 and 4.5 months of age.

### Tumor counts and measurements

At necropsy (performed at 6 months of age), coli were flushed with sterile phosphate-buffered saline, incised longitudinally, and arranged on bibulous paper for fixation in formalin. Fixed coli were pinned down on a white surface to allow full view of the mucosal surface. Grossly visible tumors were imaged using a Leica M165FC microscope equipped with a Leica DFC450 camera at 1× magnification (Leica Camera AG, Solms, Germany). Using the Leica Application Suite 4.2, the maximum perimeter of each tumor was manually outlined and the cross-sectional tumor area was calculated using the “area-line tool”.

### Statistical analysis

All statistical analyses were performed using the R software platform with specialty Bioconductor packages as given, unless otherwise noted. For all OTU comparisons, data from each time point was normalized and analyzed separately. Alpha diversity was determined via both the Chao1 and Shannon methods using the raw, full data set (without pruning OTUs), and was visualized via the phyloseq package [47]. Read count tables produced by Qiime were normalized using a scaling-factor approach (cumNorm) with a data-driven target quantile as implemented in the metagenomeSeq package [48]. cumNorm corrects for varying sequence depth across samples, and ensures all samples have the same total number of reads. Hierarchical clustering, using log_2_-transformed normalized read counts with one read added to all counts to avoid an undefined log, was performed using Euclidean distance with agglomeration based on complete linkage. Testing for interactions and main effects was performed via a mixture-model approach that estimates the probability that an observed zero count (for a given OTU and sample) is a technical zero (caused by lack of depth) or not. This models a zero-inflated Gaussian mixture distribution and has been shown to perform best in an independent comparison against other techniques in settings similar to ours (and was the only method capable of dealing with factorial designs) [49]. Prior to statistical testing at each time point, independent filtering of OTUs was performed to improve power [50]. *p*-values for testing of OTUs were adjusted for multiple testing [51]. Spearman's correlation was used to determine the correlation coefficient between tumor burden (tumor count and tumor area) and the relative abundance of selected taxa at each time point.

## SUPPLEMENTARY MATERIAL FIGURES AND TABLE



## References

[1] Claesson MJ, Jeffery IB, Conde S, Power SE, O'Connor EM, Cusack S, Harris HM, Coakley M, Lakshminarayanan B, O'Sullivan O, Fitzgerald GF, Deane J, O'Connor M (2012). Gut microbiota composition correlates with diet and health in the elderly. Nature.

[2] Wu GD, Chen J, Hoffmann C, Bittinger K, Chen YY, Keilbaugh SA, Bewtra M, Knights D, Walters WA, Knight R, Sinha R, Gilroy E, Gupta K (2011). Linking long-term dietary patterns with gut microbial enterotypes. Science.

[3] Queipo-Ortuno MI, Boto-Ordonez M, Murri M, Gomez-Zumaquero JM, Clemente-Postigo M, Estruch R, Cardona Diaz F, Andres-Lacueva C, Tinahones FJ (2012). Influence of red wine polyphenols and ethanol on the gut microbiota ecology and biochemical biomarkers. The American journal of clinical nutrition.

[4] Biedermann L, Zeitz J, Mwinyi J, Sutter-Minder E, Rehman A, Ott SJ, Steurer-Stey C, Frei A, Frei P, Scharl M, Loessner MJ, Vavricka SR, Fried M (2013). Smoking cessation induces profound changes in the composition of the intestinal microbiota in humans. PloS one.

[5] Ley RE, Backhed F, Turnbaugh P, Lozupone CA, Knight RD, Gordon JI (2005). Obesity alters gut microbial ecology. Proc Natl Acad Sci USA.

[6] Lochhead P, Chan AT, Nishihara R, Fuchs CS, Beck AH, Giovannucci E, Ogino S (2015). Etiologic field effect: reappraisal of the field effect concept in cancer predisposition and progression. Mod Pathol.

[7] Scanlan PD, Shanahan F, Clune Y, Collins JK, O'Sullivan GC, O'Riordan M, Holmes E, Wang Y, Marchesi JR (2008). Culture-independent analysis of the gut microbiota in colorectal cancer and polyposis. Environmental microbiology.

[8] Chen W, Liu F, Ling Z, Tong X, Xiang C (2012). Human intestinal lumen and mucosa-associated microbiota in patients with colorectal cancer. PloS one.

[9] Chu FF, Esworthy RS, Chu PG, Longmate JA, Huycke MM, Wilczynski S, Doroshow JH (2004). Bacteria-induced intestinal cancer in mice with disrupted Gpx1 and Gpx2 genes. Cancer Res.

[10] Kado S, Uchida K, Funabashi H, Iwata S, Nagata Y, Ando M, Onoue M, Matsuoka Y, Ohwaki M, Morotomi M (2001). Intestinal microflora are necessary for development of spontaneous adenocarcinoma of the large intestine in T-cell receptor beta chain and p53 double-knockout mice. Cancer Res.

[11] Li Y, Kundu P, Seow SW, de Matos CT, Aronsson L, Chin KC, Karre K, Pettersson S, Greicius G (2012). Gut microbiota accelerate tumor growth via c-jun and STAT3 phosphorylation in APCMin/+ mice. Carcinogenesis.

[12] Engle SJ, Ormsby I, Pawlowski S, Boivin GP, Croft J, Balish E, Doetschman T (2002). Elimination of colon cancer in germ-free transforming growth factor beta 1-deficient mice. Cancer Res.

[13] Mai V, Colbert LH, Perkins SN, Schatzkin A, Hursting SD (2007). Intestinal microbiota: a potential diet-responsive prevention target in ApcMin mice. Molecular carcinogenesis.

[14] Dove WF, Clipson L, Gould KA, Luongo C, Marshall DJ, Moser AR, Newton MA, Jacoby RF (1997). Intestinal neoplasia in the ApcMin mouse: independence from the microbial and natural killer (beige locus) status. Cancer Res.

[15] Amos-Landgraf JM, Kwong LN, Kendziorski CM, Reichelderfer M, Torrealba J, Weichert J, Haag JD, Chen KS, Waller JL, Gould MN, Dove WF (2007). A target-selected Apc-mutant rat kindred enhances the modeling of familial human colon cancer. Proc Natl Acad Sci USA.

[16] Amos-Landgraf JM, Heijmans J, Wielenga MC, Dunkin E, Krentz KJ, Clipson L, Ederveen AG, Groothuis PG, Mosselman S, Muncan V, Hommes DW, Shedlovsky A, Dove WF (2014). Sex disparity in colonic adenomagenesis involves promotion by male hormones, not protection by female hormones. Proc Natl Acad Sci USA.

[17] Candela M, Turroni S, Biagi E, Carbonero F, Rampelli S, Fiorentini C, Brigidi P (2014). Inflammation and colorectal cancer, when microbiota-host mutualism breaks. World journal of gastroenterology : WJG.

[18] Allen-Vercoe E, Jobin C (2014). Fusobacterium and Enterobacteriaceae: Important players for CRC?. Immunology letters.

[19] Arthur JC, Gharaibeh RZ, Muhlbauer M, Perez-Chanona E, Uronis JM, McCafferty J, Fodor AA, Jobin C (2014). Microbial genomic analysis reveals the essential role of inflammation in bacteria-induced colorectal cancer. Nature communications.

[20] Reddy BS, Weisburger JH, Narisawa T, Wynder EL (1974). Colon carcinogenesis in germ-free rats with 1,2-dimethylhydrazine and N-methyl-n'-nitro-N-nitrosoguanidine. Cancer Res.

[21] Scharek L, Hartmann L, Heinevetter L, Blaut M (2000). Bifidobacterium adolescentis modulates the specific immune response to another human gut bacterium, Bacteroides thetaiotaomicron, in gnotobiotic rats. Immunobiology.

[22] Rath HC, Herfarth HH, Ikeda JS, Grenther WB, Hamm TE, Balish E, Taurog JD, Hammer RE, Wilson KH, Sartor RB (1996). Normal luminal bacteria, especially Bacteroides species, mediate chronic colitis, gastritis, and arthritis in HLA-B27/human beta2 microglobulin transgenic rats. J Clin Invest.

[23] Horie H, Kanazawa K, Okada M, Narushima S, Itoh K, Terada A (1999). Effects of intestinal bacteria on the development of colonic neoplasm: an experimental study. European journal of cancer prevention : the official journal of the European Cancer Prevention Organisation.

[24] Arthur JC, Perez-Chanona E, Muhlbauer M, Tomkovich S, Uronis JM, Fan TJ, Campbell BJ, Abujamel T, Dogan B, Rogers AB, Rhodes JM, Stintzi A, Simpson KW (2012). Intestinal inflammation targets cancer-inducing activity of the microbiota. Science.

[25] Hoffmann M, Kim SC, Sartor RB, Haller D (2009). Enterococcus faecalis strains differentially regulate Alix/AIP1 protein expression and ERK 1/2 activation in intestinal epithelial cells in the context of chronic experimental colitis. Journal of proteome research.

[26] Onoue M, Kado S, Sakaitani Y, Uchida K, Morotomi M (1997). Specific species of intestinal bacteria influence the induction of aberrant crypt foci by 1,2-dimethylhydrazine in rats. Cancer Lett.

[27] Wang T, Cai G, Qiu Y, Fei N, Zhang M, Pang X, Jia W, Cai S, Zhao L (2012). Structural segregation of gut microbiota between colorectal cancer patients and healthy volunteers. ISME J.

[28] Sobhani I, Tap J, Roudot-Thoraval F, Roperch JP, Letulle S, Langella P, Corthier G, Tran Van Nhieu J, Furet JP (2011). Microbial dysbiosis in colorectal cancer (CRC) patients. PloS one.

[29] Marchesi JR, Dutilh BE, Hall N, Peters WH, Roelofs R, Boleij A, Tjalsma H (2011). Towards the human colorectal cancer microbiome. PloS one.

[30] Weir TL, Manter DK, Sheflin AM, Barnett BA, Heuberger AL, Ryan EP (2013). Stool microbiome and metabolome differences between colorectal cancer patients and healthy adults. PloS one.

[31] Castellarin M, Warren RL, Freeman JD, Dreolini L, Krzywinski M, Strauss J, Barnes R, Watson P, Allen-Vercoe E, Moore RA, Holt RA (2012). Fusobacterium nucleatum infection is prevalent in human colorectal carcinoma. Genome Res.

[32] Kostic AD, Gevers D, Pedamallu CS, Michaud M, Duke F, Earl AM, Ojesina AI, Jung J, Bass AJ, Tabernero J, Baselga J, Liu C, Shivdasani RA (2012). Genomic analysis identifies association of Fusobacterium with colorectal carcinoma. Genome Res.

[33] Wu N, Yang X, Zhang R, Li J, Xiao X, Hu Y, Chen Y, Yang F, Lu N, Wang Z, Luan C, Liu Y, Wang B (2013). Dysbiosis signature of fecal microbiota in colorectal cancer patients. Microb Ecol.

[34] Balamurugan R, Rajendiran E, George S, Samuel GV, Ramakrishna BS (2008). Real-time polymerase chain reaction quantification of specific butyrate-producing bacteria, Desulfovibrio and Enterococcus faecalis in the feces of patients with colorectal cancer. Journal of gastroenterology and hepatology.

[35] Shen XJ, Rawls JF, Randall T, Burcal L, Mpande CN, Jenkins N, Jovov B, Abdo Z, Sandler RS, Keku TO (2010). Molecular characterization of mucosal adherent bacteria and associations with colorectal adenomas. Gut microbes.

[36] Hinnebusch BF, Meng S, Wu JT, Archer SY, Hodin RA (2002). The effects of short-chain fatty acids on human colon cancer cell phenotype are associated with histone hyperacetylation. The Journal of nutrition.

[37] Kiefer J, Beyer-Sehlmeyer G, Pool-Zobel BL (2006). Mixtures of SCFA, composed according to physiologically available concentrations in the gut lumen, modulate histone acetylation in human HT29 colon cancer cells. The British journal of nutrition.

[38] Kang DW, Park JG, Ilhan ZE, Wallstrom G, Labaer J, Adams JB, Krajmalnik-Brown R (2013). Reduced incidence of Prevotella and other fermenters in intestinal microflora of autistic children. PloS one.

[39] Arumugam M, Raes J, Pelletier E, Le Paslier D, Yamada T, Mende DR, Fernandes GR, Tap J, Bruls T, Batto JM, Bertalan M, Borruel N, Casellas F (2011). Enterotypes of the human gut microbiome. Nature.

[40] Hambly RJ, Rumney CJ, Cunninghame M, Fletcher JM, Rijken PJ, Rowland IR (1997). Influence of diets containing high and low risk factors for colon cancer on early stages of carcinogenesis in human flora-associated (HFA) rats. Carcinogenesis.

[41] Venturi M, Hambly RJ, Glinghammar B, Rafter JJ, Rowland IR (1997). Genotoxic activity in human faecal water and the role of bile acids: a study using the alkaline comet assay. Carcinogenesis.

[42] Maurice CF, Haiser HJ, Turnbaugh PJ (2013). Xenobiotics shape the physiology and gene expression of the active human gut microbiome. Cell.

[43] McKnite AM, Perez-Munoz ME, Lu L, Williams EG, Brewer S, Andreux PA, Bastiaansen JW, Wang X, Kachman SD, Auwerx J, Williams RW, Benson AK, Peterson DA (2012). Murine gut microbiota is defined by host genetics and modulates variation of metabolic traits. PloS one.

[44] Jiao S, Peters U, Berndt S, Brenner H, Butterbach K, Caan BJ, Carlson CS, Chan AT, Chang-Claude J, Chanock S, Curtis KR, Duggan D, Gong J (2014). Estimating the heritability of colorectal cancer. Hum Mol Genet.

[45] Ericsson AC, Davis JW, Spollen W, Bivens N, Givan S, Hagan CE, McIntosh M, Franklin CL (2015). Effects of vendor and genetic background on the composition of the fecal microbiota of inbred mice. PloS one.

[46] Irving AA, Young LB, Pleiman JK, Konrath MJ, Marzella B, Nonte M, Cacciatore J, Ford MR, Clipson L, Amos-Landgraf JM, Dove WF (2014). A simple, quantitative method using alginate gel to determine rat colonic tumor volume in vivo. Comp Med.

[47] McMurdie PJ, Holmes S (2013). phyloseq: an R package for reproducible interactive analysis and graphics of microbiome census data. PloS one.

[48] Paulson JN, Pop M, Bravo HC metagenomeSeq: Statistical analysis for sparse high-throughput sequencing. Bioconductor package 160. http://cbcb.umd.edu/software/metagenomeSeq.

[49] Paulson JN, Stine OC, Bravo HC, Pop M (2013). Differential abundance analysis for microbial marker-gene surveys. Nature methods.

[50] Bourgon R, Gentleman R, Huber W (2010). Independent filtering increases detection power for high-throughput experiments. Proc Natl Acad Sci USA.

[51] Benjamini Y HY (1995). Controlling the false discovery rate: a practical and powerful approach to multiple testing. Journal of the Royal Statistical Society Series B.

