# Supplementary material for: Differential susceptibility to colorectal cancer due to naturally occurring gut microbiota
Source: Oncotarget. 2015 Sep 10;6(32):33689–704. doi: 10.18632/oncotarget.5604 (PMC4741795; doi:10.18632/oncotarget.5604)
Supplement: Supplementary file 1 [file oncotarget-06-33689-s001.pdf]

Differential susceptibility to colorectal cancer due to naturally occurring gut microbiota

Supplementary Material

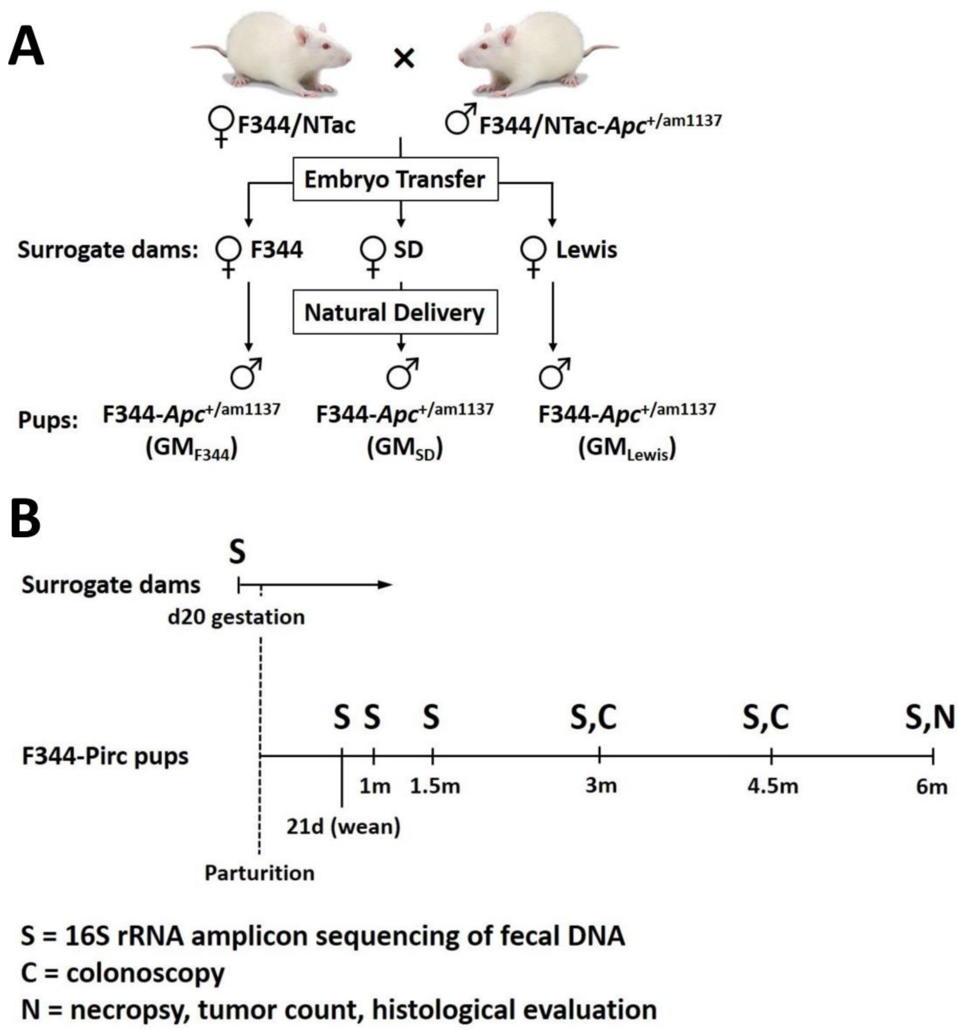

Supplementary Fig. S1: Experimental design used to generate isogenic F344-Pirc rats with different gut microbiota. Schematic showing the breeding and embryo transfer scheme used to generate isogenic F344-Pirc rats harboring different gut microbiota profiles (**A**), and timeline depicting sample collection and procedural time points (**B**).

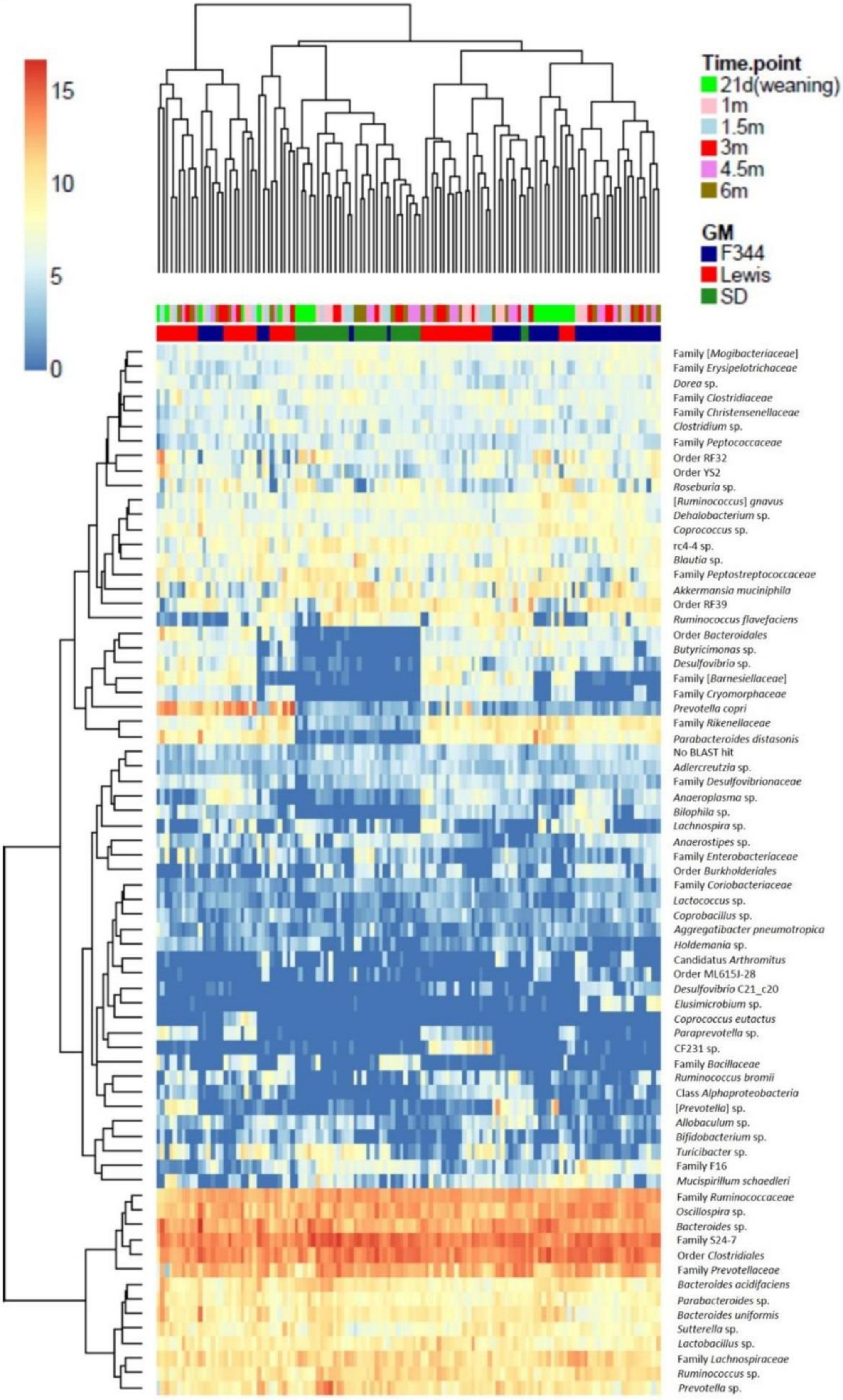

15
10
5
0
Time.point
21d(weaning)
1m
1.5m
3m
4.5m
6m
GM
F344
Lewis
SD
Family [Mogibacteriaceae]
Family Erysipelotrichaceae
Dorea sp.
Family Clostridiaceae
Family Christensenellaceae
Clostridium sp.
Family Peptococcaceae
Order RF32
Order YS2
Roseburia sp.
[Ruminococcus] gnavus
Dehalobacterium sp.
Coprococcus sp.
rc4-4 sp.
Blautia sp.
Family Peptostreptococcaceae
Akkermansia muciniphila
Order RF39
Ruminococcus flavefaciens
Order Bacteroidales
Butyricimonas sp.
Desulfovibrio sp.
Family [Barnesiellaceae]
Family Cryomorphaceae
Prevotella copri
Family Rikenellaceae
Parabacteroides distasonis
No BLAST hit
Adlercreutzia sp.
Family Desulfovibrionaceae
Anaeroplasma sp.
Bilophila sp.
Lachnospira sp.
Anaerostipes sp.
Family Enterobacteriaceae
Order Burkholderiales
Family Coriobacteriaceae
Lactococcus sp.
Coprobacillus sp.
Aggregatibacter pneumotropica
Holdemania sp.
Candidatus Arthromitus
Order ML615J-28
Desulfovibrio C21_c20
Elusimicrobium sp.
Coprococcus eutactus
Paraprevotella sp.
CF231 sp.
Family Bacillaceae
Ruminococcus bromii
Class Alphaproteobacteria
[Prevotella] sp.
Allobaculum sp.
Bifidobacterium sp.
Turicibacter sp.
Family F16
Mucispirillum schaedleri
Family Ruminococcaceae
Oscillospira sp.
Bacteroides sp.
Family S24-7
Order Clostridiales
Family Prevotellaceae
Bacteroides acidifaciens
Parabacteroides sp.
Bacteroides uniformis
Sutterella sp.
Lactobacillus sp.
Family Lachnospiraceae
Ruminococcus sp.
Prevotella sp.

Supplementary Fig. S2: **Hierarchical cluster analysis of male F344-Pirc rats.** Agglomerative hierarchical clustering analysis of the 71 consistently detected operational taxonomic units (OTUs) in the GM of male F344-Pirc pups born to F344, SD, or Lewis surrogate dams, at weaning (21d), 1 month (1m), 1.5m, 3m, 4.5m, and 6m of age. Color intensity shows $\log_2$-transformed normalized abundance of OTUs in each sample (bar at upper left). Operational taxonomic units are listed at right, time of sampling and identity of surrogate dam are denoted by color-coded bars at top (legend at upper right).

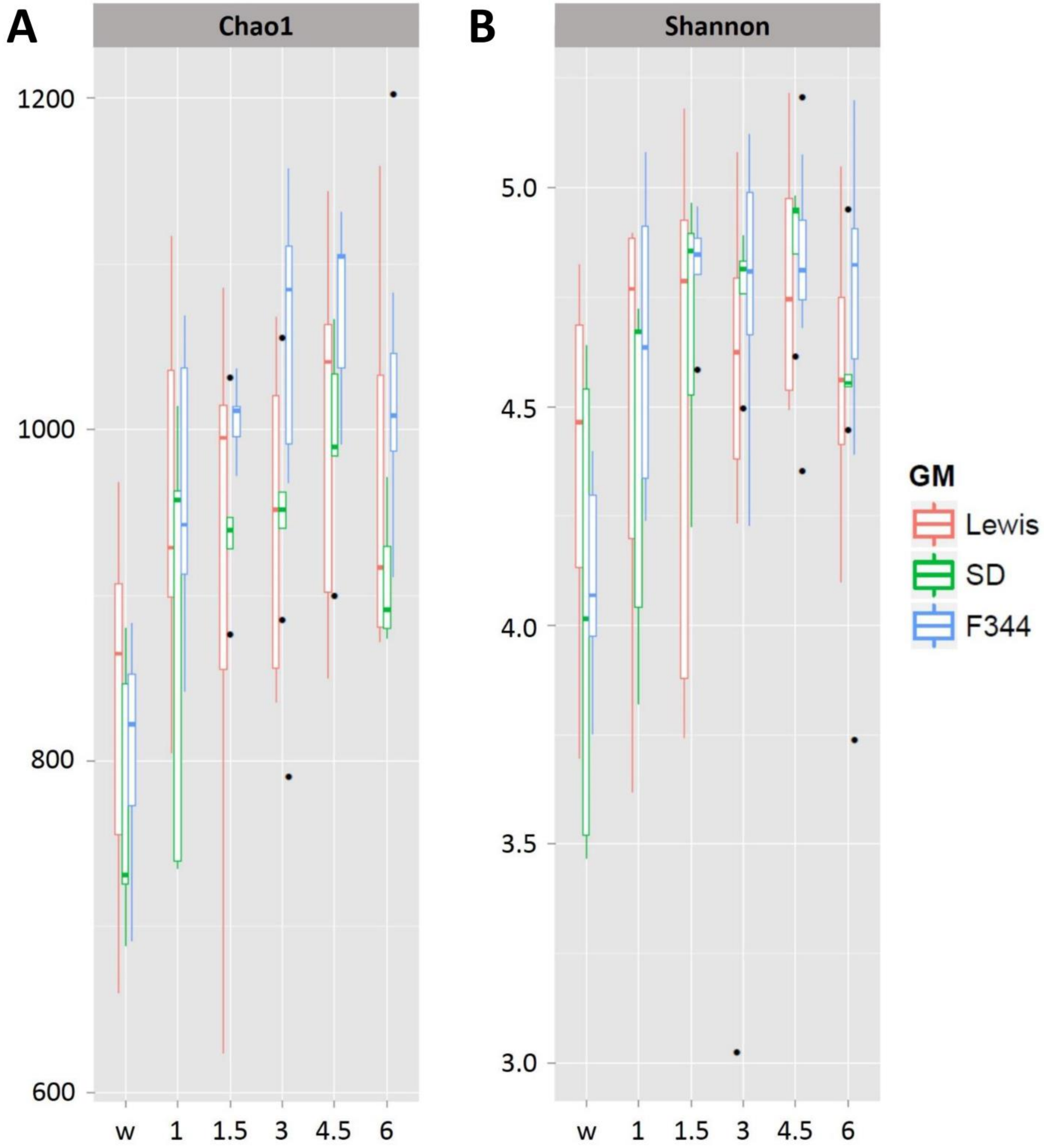

Supplementary Fig. S3: **Diversity of fecal microbiota.** Box plots of Chao1 (**A**) and Shannon (**B**) estimates of microbial diversity plotted by time point. Data points are colored to indicate GM profile (GM_{F344}, blue; GM_{SD}, green; GM_{Lewis}, red).

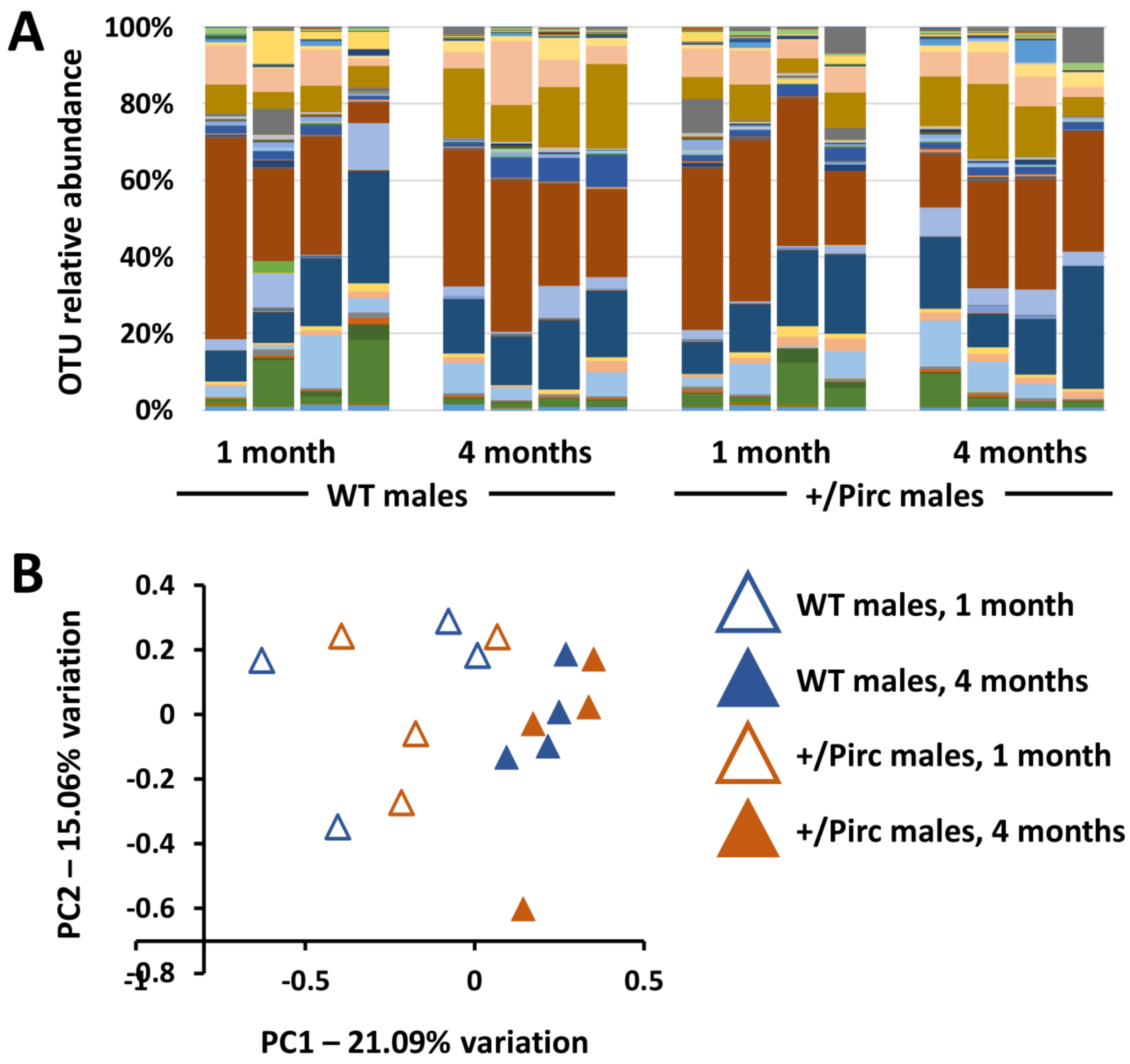

Supplementary Fig. S4: Influence of the +/Pirc genotype on the gut microbiota in male rats. Bar charts showing the relative abundance of operational taxonomic units (OTUs) detected in the feces of non-rederived male wild-type (WT) and heterozygous (+/Pirc) littermates (*n* = 4/group), collected at 1 and 4 months of age (**A**); principal component analysis of the samples shown in A, demonstrating clustering of samples by age, rather than genotype (**B**; legend at right).

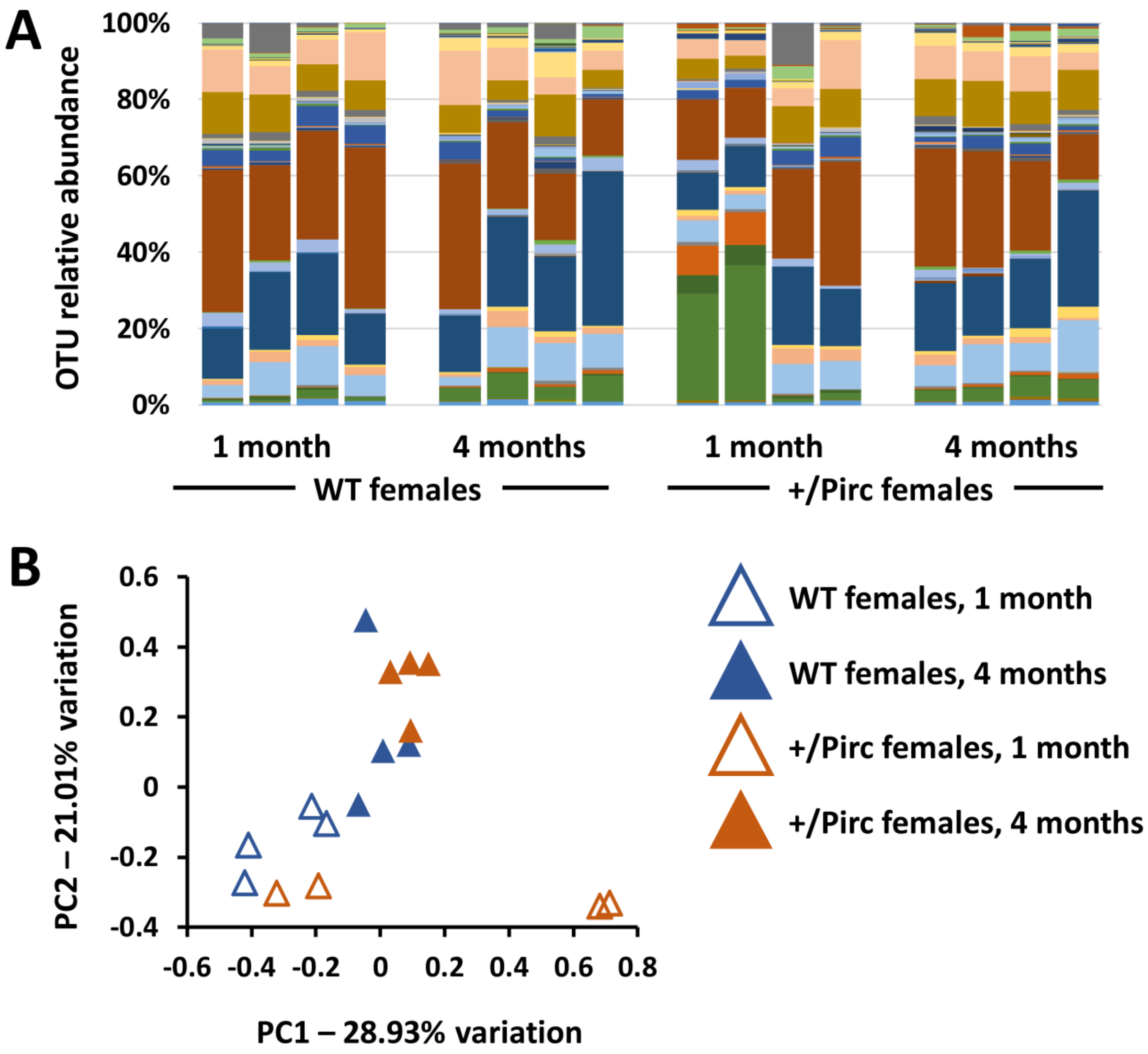

Supplementary Fig. S5: Influence of the +/Pirc genotype on the gut microbiota in female rats. Bar charts showing the relative abundance of operational taxonomic units (OTUs) detected in the feces of non-rederived female wild-type (WT) and heterozygous (+/Pirc) littermates (*n* = 4/group), collected at 1 and 4 months of age (**A**); principal component analysis of the samples shown in A, demonstrating clustering of samples by age, rather than genotype (**B**; legend at right).

Supplementary Table 1: Summary of rederivation attempts resulting in F344-Pirc offspring. Number and genotype of male and female offspring born from each round of embryo transfer into F344/NHsd (F344), Crl:SD (SD), and LEW/SsNHsd (Lewis) surrogate dams. ID = dam ear-tag number, DOB = date of birth.

Surrogate dams				Male pups		Female pups	
Background	ID	DOB	# pups	WT	+/Pirc	WT	+/Pirc
F344/NHsd	270RA	9/12/13	3	0	1	unknown	
	049RG	9/17/13	5	0	1	unknown	
	795RC	10/23/13	11	4	2	unknown	
	349RJ	12/11/13	12	3	1	unknown	
	649RJ	2/19/14	11	2	3	unknown	
Crl:SD	794RC	10/23/13	11	1	5	1	4
LEW/SsNHsd	235RH	8/27/13	7	2	1	unknown	
	100RH	9/3/13	10	3	1	unknown	
	050RG	9/17/13	6	0	2	unknown	
	424RD	10/16/13	13	0	3	5	5
	131RH	10/30/13	6	2	1	1	2
